# Adjuvant radiotherapy and chemotherapy for patients with breast phyllodes tumors: a systematic review and meta-analysis

**DOI:** 10.1186/s12885-019-5585-5

**Published:** 2019-04-23

**Authors:** Xue Chao, Kai Chen, Jiayi Zeng, Zhuofei Bi, Mingyan Guo, Yi Chen, Yandan Yao, Wei Wu, Shi Liang, Yan Nie

**Affiliations:** 1 Guangdong Provincial Key Laboratory of Malignant Tumor Epigenetics and Gene Regulation, Sun Yat-Sen Memorial Hospital, Sun Yat-Sen University, 107 Yanjiang West Road, Guangzhou, 510120 People’s Republic of China; 2 Breast Tumor Center, Sun Yat-Sen Memorial Hospital, Sun Yat-Sen University, 107 Yanjiang West Road, Guangzhou, 510120 People’s Republic of China; 3 Guangzhou Zhixin High School, 152 Zhixin South Road, Guangzhou, 510120 People’s Republic of China; 4 Department of Radiation Oncology, Sun Yat-Sen Memorial Hospital, Sun Yat-Sen University, 107 Yanjiang West Road, Guangzhou, 510120 People’s Republic of China; 5 Department of Anesthesiology, Sun Yat-sen Memorial Hospital, Sun Yat-sen University, 107 Yanjiang West Road, Guangzhou, 510120 People’s Republic of China

**Keywords:** Phyllodes tumors, Radiotherapy, Chemotherapy, Meta-analysis

## Abstract

**Background:**

As the efficacy of radiotherapy and chemotherapy for treatment of phyllodes tumors (PTs) remains unclear, this study aimed to review all available data and evaluate the roles of radiotherapy and chemotherapy in PT treatment.

**Methods:**

We performed a comprehensive search of databases, including PubMed, Web of Science and the Cochrane Library. The outcomes of interest included the local recurrence (LR) rate, metastasis rate, disease-free survival rate and overall survival rate.

**Results:**

Seventeen studies enrolling 696 patients were included in this random effect meta-analysis. Subgroup analysis and meta-regression were also conducted to determine study heterogeneity. A pooled local recurrence rate of 8% (95% CI: 1–22%) was observed with a statistical heterogeneity of I^2^ = 86.6% (*p* < 0.01) for radiotherapy. This was lower than the recurrence rate of 12% for simple surgical treatment (95% CI: 7–18%). Meta-regression analysis found that surgical margin status was the main source of heterogeneity (*p* = 0.04). The metastasis rate of 4% (95% CI: 0–11%) for patients receiving radiotherapy without significant heterogeneity was also lower than the rate for the simple surgery group (8, 95% CI: 3–15%). The available data for chemotherapy were too limited to support meta-analysis. Accordingly, we offer a pure review of these data.

**Conclusion:**

Our findings suggest that radiotherapy is effective in achieving local disease control and preventing metastasis.

**Electronic supplementary material:**

The online version of this article (10.1186/s12885-019-5585-5) contains supplementary material, which is available to authorized users.

## Background

Phyllodes tumors (PTs) of the breast are typically large, rapidly growing tumors that account for up to 1% of all breast neoplasms [1]. The World Health Organization (WHO) classifies phyllodes tumors into three histologic subtypes: benign, borderline, and malignant, based on stromal cellularity, stromal cell mitotic activity, stromal nuclear atypia, stromal overgrowth and type of borders (infiltrating or pushing) [2–4].

Although many phyllodes tumors tend to behave in a benign manner, the clinical outcomes of phyllodes tumors are hard to predict because of relatively high recurrence rates and occasional distant metastases. The current approach to preventing local relapse and metastasis is surgical resection with wide margins. However, even with wide surgical resection, the local recurrence rate remains as high as 8 to 36% [5]. Furthermore, recurrent phyllodes tumors can progress toward more malignant phenotypes [6], in which metastases have been estimated to occur in up to 25% of patients [7].

Despite these data, no well-established adjuvant therapy applies to high grade phyllodes tumors, which is partly due to the controversial roles of adjuvant radiotherapy and chemotherapyl [8]. The use of radiotherapy lacks sufficient prospective study data regarding borderline and malignant PT [9], while the adoption of chemotherapy is yet to solve the rarity of disease presentation [10]. Furthermore, no randomized clinical trials of chemotherapy and/or radiation therapy have been published.

To help address this problem, we performed a literature review and meta-analysis to evaluate the roles of radiotherapy and chemotherapy in PT treatment.

## Methods

### Search strategy

We performed a comprehensive search of databases, including PubMed, Web of Science, Embase and the Cochrane Library, from 1985 to Feb 1, 2019. The following MeSH terms and their combinations were searched: (breast tumor/ sarcoma/ neoplasm) and (phyllodes or phyllode) and (radiotherapy/ chemotherapy).

### Inclusion and exclusion criteria

Articles concerning radiotherapy and chemotherapy as adjuvant therapy in women with breast phyllodes tumors were included, regardless of prospective or retrospective ones. However, case reports were excluded. So were studies without the outcomes of interest, such as local recurrence, survival and distant metastasis.

### Data extraction

Data were extracted independently by two of the authors (N.Y. and C.X). Consensus was reached between the two authors if there was a discrepancy among the collected data. For each included study, the following information was collected: author, year of publication, country, months of follow-up, number of total cases and patients treated with adjuvant therapy, surgery type and percentage, type and details of adjuvant therapy, age, tumor size, histological type, margins, number of local recurrences, number of metastases, number of patients surviving disease-free and number of overall survivors. A quality assessment of the included studies was also performed based on the tool for case series studies provided by U.S. Department of Health & Human Services (https://www.nhlbi.nih.gov/health-topics/study-quality-assessment-tools). Per study, there were many aspects of study design that required termwise evaluation, including objective of the study, cases definition, subjects, interventions, outcome measures, follow-up, statistical methods and results. Quality reviewers could select “yes,” “no,” or “cannot determine/not reported/not applicable” in response to each item on the tool. For each item where “no” was selected, reviewers were instructed to consider the potential risk of bias that could be introduced by that flaw in the study design or implementation. Cannot determine and not reported were also noted as representing potential flaws. The procedure was done independently by two reviewerss (X.C and K.C.). A review grade (good, fair or poor) was allocated to each study. A “good” study has the least risk of bias, and results are considered to be valid. A “fair” study is susceptible to some bias deemed not sufficient to invalidate its results. A “poor” rating indicates significant risk of bias.

### Meta-analysis

As the data included in our study were all either < 0.2 or > 0.8, these data do not follow a normal distribution, and therefore continuous outcome data could not be carried out. The Free-Tukey double arcsine transformation transformed the data to follow a normal distribution, which then could be used for meta-analysis. Once the meta-analysis is done, the output data were back-transformed and therefore through this process we can compare the data obtained via meta-analysis with original data [11]. Separate analysis was performed based on the transformed proportions using random models. All data were back-transformed to determine the rate and 95% confidence interval (CI). Statistical heterogeneity among studies was assessed using Q and I^2^ statistics. For the Q statistics, data were heterogeneous if *p* < 0.1. The Q test was used to test for effect size heterogeneity. I^2^ values of < 25, 25–75% and > 75% corresponded to low, moderate, and high degrees of heterogeneity, respectively. Sensitivity analysis and meta-regression analyses were conducted to determine the origin of heterogeneity. Meta-regression analysis was achieved by using liner regression models. We also conducted subgroup analyses based on study size, surgery type, age, tumor size, histological type and margins. All statistical analyses were performed by using Stata 13.0 (Stata, College Station, Texas, USA).

## Results

### Search results

**Fig. 1:**
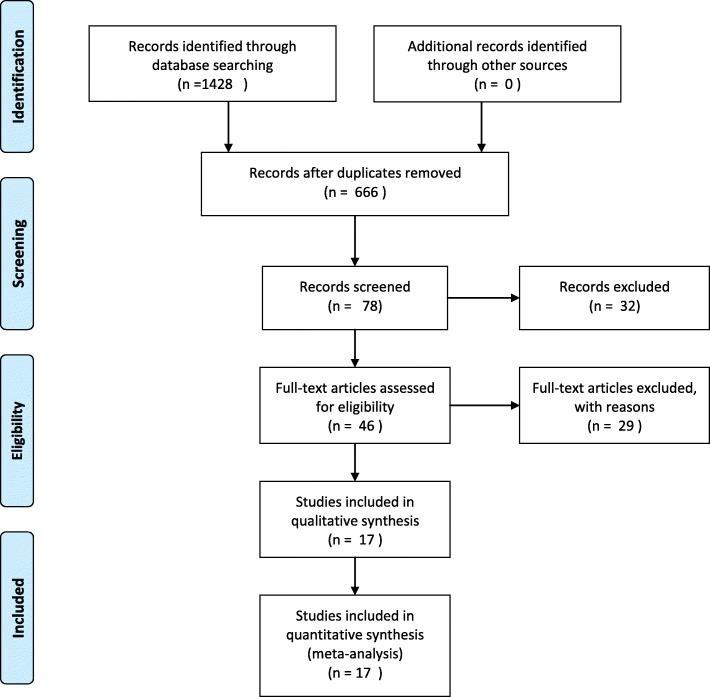
Flow diagram of the literature search

The search strategy identified 337 studies of radiotherapy and 329 studies of chemotherapy. After a thorough review of abstracts and exclusion of duplicated references, 46 studies were treated as candidates. Further examination of the manuscripts led to the inclusion of 17 radiotherapy studies for meta-analysis [3, 9, 12–26]. The literature search flow chart is displayed in Fig. 1. A total of 696 patients were included in the study. The detailed characteristics of the included studies are shown in Table 1. The qualities of the included studies were all rated as good. And the detail of the quality assessment was displayed in Additional file 2: Table S1. Unfortunately, the small number and scale of studies on chemotherapy for PT prevented meta-analysis of the effectiveness of chemotherapy. Nevertheless, studies concerning chemotherapy are listed in Table 2 [3, 8, 10, 20, 27].

**Table 1:** Characteristics of studies involved in radiotherapy of PT treatment

Author	Year	Countr	Follow-up	No. case	Radio	Surgery Type(%)		Age	Tumor size (cm)	Histological Type(%)			Margin(%)		LR	DFS	OS	MS
						BCS	M			Be	Bo	Ma	< 1 cm (positive)	➢ 1 cm (negative)				
Barth	2009	Lebano	56 m	46	46	100	0	49 (mean)	3.7(mean)	/	34.8	63.2	34.8	63.2	0	46	44	2
Belkacemi	2008	France	106 m	443	39	85.1	14.9	40 (median)	3(median)	64.1	18.1	17.8	15	43	4	26	34	/
Chaney	1998	USA	36.5 m	8	8	12.5	87.5	43 (median)	10.4(median	12.5	25	62.5	25	75	0	8	8	/
Chaney	2000	USA	47 m	101	6	47	53	41 (median)	4	58.4	11.8	30	(0.1)	(99.9)	0	6	5	/
Chen, W	2005	Taiwan	71 m	172	2	6.4	93.6	37 (mean)	5.8(mean)	76.1	6.9	17	(7.5)	(92.5)	0	/	2	2
Cheng	2006	Taiwan	30 m	81	2	72.8	27.2	37(mean)	7.7	72.8	7.4	19.8	(6.2)	(93.8)	0	/	2	0
Choi	2018	Korea	5 yrs	362	31	73.2	26.8	43(median)	6(mean)	/	64.9	35.1	(10.4)	(89.6)	1	30	31	0
Cohn-Ce	1991	Sweden	8 yr	77	24	40.2	59.8	50(median)	NA	51.9	0	48.1	/	/	13	/	/	3
Demian	2016	Egypt	52 m	35	4	57	43	40(median)	6.8	3	37	60	(20)	(80)	0	4	4	0
Gnerlic	2014	USA	53 m	3120	446	56	44	51.1(mean)	4.2(median)	/	/	100	(10)	(90)	132	/	/	/
Guillot	2011	France	12.65 m	165	8	58.7	41.3	44(median)	3	65.5	21.8	12.7	27.8	71.2	2	/	6	/
Joshi,	2003	India	35 m	26	4	46.2	53.8	38(median)	6(mean)	65.4	11.5	23.1	(15.4)	(84.6)	0	4	/	0
Liew, K. W.	2018	Sabah	11 m	11	6	36	64	45(median)	10.5(median)	0	0	100	(27)	(73)	4	2	6	0
Mitus	2019	Poland	12 yrs	340	12	100	0	51(mean)	6(mean)	/	33	67	100	0	0	12	12	0
Park, H. J.	2019	Korea	76 m	43	43	61.4	37.6	42(median)	5.8(median)	0	0	100	(9)	(91)	0	37	37	6
Stranzl	2004	Austri	33.8 m	6	6	/	100	53(median)	7(median)	/	33	67	67	33	/	4	5	0
Varghese	2017	India	20 m	92	9	51.1	48.9	43(median)	10(median)	60	23	17	/	/	1	8	9	0

*BCS* breast conserving surgery, *M* mastectomy, *Be* benign, *Bo* borderline, *Ma* malignant, *LR* local recurrence, *DFS* disease-free survival, *OS* overall survival *MS metastasis*

**Table 2:** Characteristics of studies involved in chemotherapy of PT treatment

Author	Year	Countr	Follow-Up	Chemo regime	No. chemoth	Surgery Type(%)		Age	Tumor Size (cm)	Histological Type(%)			Margin		LR	DFS	OS	MS
						BCS	M			Be	Bo	M	< 1 cm (%) (positive)	> 1 cm (%) (negative)				
Chaney,	2000	USA	47 m	doxorubicin- ifosfamide ifosfamide 4300 mg	4	47	53	41(median)	4	/	20	80	(1)	(99)	0	0	4	4
Guillot,	2011	France	12.65 m	Adriamycin 100 mg *6	9	97	3	44(median)	3	/	/	100	28	72	2	6	6	2
Morales	2007	Mexico	15 m	Doxorubicin + dacarbazine	17	23.5	76.5	42(median)	13	/	/	100	(24)	(76)	6	/	14	10
Wang, F.	2014	China	NA	NA	8	61.4	38.6	49(mean)	5	/	/	100	NA	NA	/	5	6	/

Chemo-Chemotherapy; *BCS* breast conserving surgery, *M* mastectomy, *Be* benign, *Bo* borderline, *LR* local recurrence, *DFS* disease-free survival, *OS* overall survival, *MS* metastasis, *NA* not available

### Meta-analysis results

#### Local recurrence

**Fig. 2:**
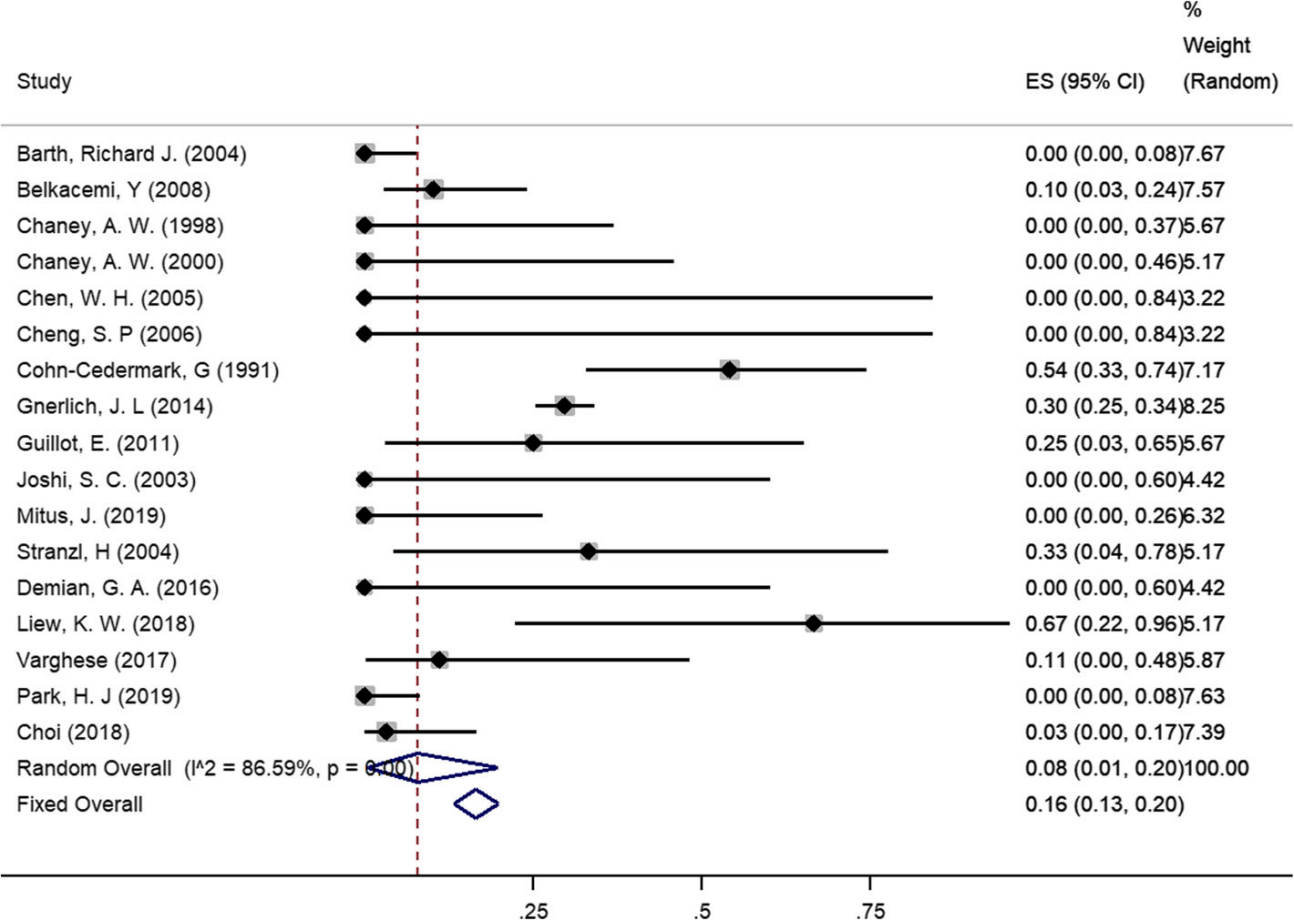
Meta-analysis of local recurrence rate of patients treated with radiotherapy (random model)

Analysis of 17 studies revealed that the group that underwent radiotherapy (Fig. 2) plus surgery had a lower local recurrence rate (8%, 95% CI: 1–22%)compared with the pooled local recurrence rate 19% (95% CI: 16–32%; test for heterogeneity: I^2^ = 24.5%, *p* = 0.19) for the group that underwent surgery alone, with statistical heterogeneity (I^2^ = 86.6%, *p* < 0.01). Meta-regression analysis revealed that the margin status of surgery accounted for 89.18% of the heterogeneity (*p* = 0.04). Subgroup analyses were also conducted based on surgery type, age, tumor size, histological type and margins. In general, the subgroup analysis showed that study size, tumor size, age and histological type may also be the causes of heterogeneity. Local recurrence rates of different histological types were also calculated. The results are listed in Table 3.

**Table 3:** Sub-group analysis of LR rate of radiotherapy + surgery

Characteristic	No. of studies	Recurrence rate (95%CI)	Heterogeneity	
			*p*	I^2^ (%)
Study size				
< 20	11	0.07(0.00–0.21)	0.08	40.5
≥ 20	6	0.11(0.00–0.30)	< 0.01	94.9
Follow-up				
< 5 yrs	11	0.10(0.00–0.34)	< 0.01	82.9
≥ 5 yrs	6	0.06(0.00–0.25)	< 0.01	87.0
Surgery type				
BCS ≥ 60%	8	0.11(0.00–0.31)	< 0.01	88.1
BCS < 60%	9	0.06(0.00–0.20)	< 0.01	65.3
Age				
< 45	11	0.01(0.00–0.04)	0.36	8.6
≥ 45	6	0.22(0.04–0.48)	< 0.01	92.6
Tumor size				
< 5 cm	5	0.18(0.02–0.42)	< 0.01	92.3
≥ 5 cm	10	0.01(0.00–0.03)	0.38	6.3
Histologic Type				
Bo + M	8	0.10(0.05–0.28)	< 0.01	93.2
Malignant	9	0.06(0.00–0.20)	0.13	35.5
Margin				
> 1 cm ≥ 50%	4	0.01(0.00–0.15)	0.08	55.5
> 1 cm < 50%	3	0.08(0.00–0.27)	0.12	53.4
Positive ≥10%	6	0.10(0.05–0.27)	0.04	57.4
Positive < 10%	3	0.05(0.00–0.45)	< 0.01	95.0

*LR* local recurrence, *BCS* Breast conserving surgery, *Bo* Borderline, *M* Malignant

#### Distant metastasis

Twelve studies were combined to reveal a metastasis rate of 4% (95% CI: 0–11%) among patients treated with radiotherapy (Additional file 1: Figure S1). There was no significant statistical heterogeneity in this analysis (I^2^ = 41.2%, *p* = 0.07). For the group undergoing surgery, the pooled metastasis rate was 8% (95% CI: 3–15%). Subgroup analyses were also conducted based on surgery type, age, tumor size, histological type and margins (Additional file 2: Table S2).

#### Disease-free survival rate and overall survival rate

Disease-free survival rate refers to the proportion of patients survives after surgery without any sign or symptom of PTs. Eleven studies were included in the analysis of disease-free survival rate at the median of the follow-up. The total disease-free survival rate was 93%(95% CI: 79–100%) with significant statistical heterogeneity (I^2^ = 76.5%, *p* < 0.01) (Additional file 3: Figure S2). Sensitivity analysis, which was performed by omitting one study at a time and then calculating the pooled disease-free survival rate for the remaining studies, showed that the study by Barth, R.J. [12] had the greatest influence on the pooled rate. After excluding this single study, the disease-free survival rate was 89% (95% CI: 72–99%, I^2^ = 39.4%, *p* = 0.16). The pooled disease-free survival rate for the surgery group was 70% (95% CI: 40–89%). The subgroup analysis is shown in Additional file 2: Table S3.

Twelve studies were analyzed to obtain a pooled overall survival rate of 96% (95% CI: 89–100%) with moderate statistical heterogeneity (I^2^ = 48.7%, *p* = 0.02). The forest plot was displayed in Additional file 4: Figure S3.Subgroup analysis suggested that tumor size, surgery type and especially histological type may be the causes of heterogeneity (Additional file 2: Table S4). We also calculated a combined overall survival rate of 76% (95% CI: 17–100%) for patients who had surgery without any adjuvant therapy.

## Discussion

PTs are divided into three types: benign, borderline and malignant, according to their histological characteristics [3]. Surgical treatment, including breast-conserving surgery and mastectomy, are the mainstays of curative treatment of PTs. However, these patients still encounter a fairly high overall recurrence rate of 19.1% [28] and a 25% malignant recurrence rate, according to a review involving 5530 patients [28]. Furthermore, malignant and borderline PT had metastasis rates ranging from 22 to 75% and mortality rates ranging from 23 to 32% [29–32]. These data emphasize the importance of local control and prevention of metastasis.

Currently, the use of adjuvant therapy for PTs remains controversial because of inadequate data from large prospective studies. The absence of these data may be due to the low incidence of PTs and the limited utilization of adjuvant therapy.

Recently, adjuvant radiotherapy has been more frequently utilized. According to a study in the National Cancer Database from the American College of Surgeons’ Commission on Cancer involving 3120 patients, adjuvant radiotherapy for PTs in 2008–2009 was used in 19.5% of cases, more than doubled compared to the rate of 9.5% in 1998–1999. [18] Data from this large retrospective study suggested that radiotherapy could extend the time to local recurrence and decrease the local recurrence rate, with no significant influence on survival. Another prospective study also discussed the effectiveness of radiotherapy for local disease control [12]. However, scant data has discussed the relationship between radiotherapy and metastasis. The pooled LR rate for patients who underwent both radiotherapy and surgery was (8%, 95% CI: 1–22%). These data were lower than both our calculated LR rate of 19% (95% CI: 7–18%) and the pooled LR rates of borderline (13%) and malignant (18%) reported by a meta-analysis [33]. Furthermore, the results of our subgroup analysis suggested that irradiation may be more effective in younger patients (< 45 years), patients with larger tumors, patients with malignant tumors and patients with wider excision. The meta-regression analysis also confirmed the importance of margin status in local control. These data emphasize the importance of ensuring adequate surgical margins. However, surgery type showed less impact on disease control based on the subgroup analysis. We suggest that for those PTs with high malignancy, radiotherapy should be used as adjuvant therapy without consideration of the surgery type. Our calculated metastasis rate of 4% (95% CI: 0–13%) in patients treated by radiotherapy, compared with a metastasis rate of 8% (95% CI: 3–15%) in patients receiving only surgical treatment, also suggested that radiotherapy may be effective in the prevention of metastasis. Although our calculated survival data (disease-free survival rate: 93%, overall survival rate: 96%) for radiotherapy are higher than those for the surgery group (disease-free survival rate rate: 70%, overall survival rate: 76%), they are similar to the data from the Surveillance, Epidemiology, and End Results database (SEER). The estimated 5-, 10-, and 15-year rates of cancer-specific survival for all women were 91, 89, and 89%, respectively [34]. This suggests that radiotherapy may have little effect on prolonging survival. Further subgroup analysis also showed improved survival among patients with cleaner margins.

Our review of the available data shows a negligible role for chemotherapy in the treatment of PTs. Most clinicians avoid chemotherapy as a first-line treatment due to lack of evidence. To date, there has been only one prospective study involving 28 patients, which has showed that chemotherapy has little effect on survival [10]. The sample sizes of other retrospective studies were also too small to prove the efficacy of chemotherapy for PTs treatment. Moreover, PTs with higher histological grades have higher metastatic potential. Few studies discuss the treatment for patients with metastatic PTs. However, the results of these few studies seem promising as nearly half of the patients exhibited partial responses to treatment [20, 35].

Our study was the first meta-analysis designed to evaluate the efficacy of radiotherapy in PTs treatment. However, there were some limitations to our study. First, we calculated only recurrence and survival rate data without considering elapsed time. Second, the studies in our meta-analysis were all observational studies, the quality of which may be sub-optimal. In conclusion, the limited incidence of PT remains a challenge for the study of PT.

## Conclusions

In summary, this study indicates that radiotherapy is effective in PTs disease control without prolonging survival. However, the data examined were mostly retrospective and permitted comparative analysis between published treatments, which is a common limitation throughout the literature. Therefore, further studies, particularly prospective studies, are needed to prove the efficacy of adjuvant therapy.

## Additional files

Additional file 1: **Figure S1**. Meta-analysis of metastasis rate of patients treated with radiotherapy (random model). (TIF 3097 kb)

Additional file 2: **Table S1.** Quality assessment of the included studies. **Table S2.** Subgroup analysis of metastasis rate of radiotherapy. **Table S3.** Subgroup analysis of disease-free survival rate. **Table S4.** Subgroup analysis of overall survival rate. (ZIP 73 kb)

Additional file 3: **Figure S2.** Meta-analysis of disease-free survival rate of patients treated with radiotherapy (random model). (TIF 1101 kb)

Additional file 4: **Figure S3**. Meta-analysis of overall survival rate of patients treated with radiotherapy (random model). (TIF 1054 kb)

## Abbreviations

CI: Confidence interval
LR: Local recurrence
PT: Phyllodes tumors
