# Supplementary figures and images for: Adjuvant radiotherapy and chemotherapy for patients with breast phyllodes tumors: a systematic review and meta-analysis
Source: BMC Cancer. 2019 Apr 23;19:372. doi: 10.1186/s12885-019-5585-5 (PMC6480723; doi:10.1186/s12885-019-5585-5)

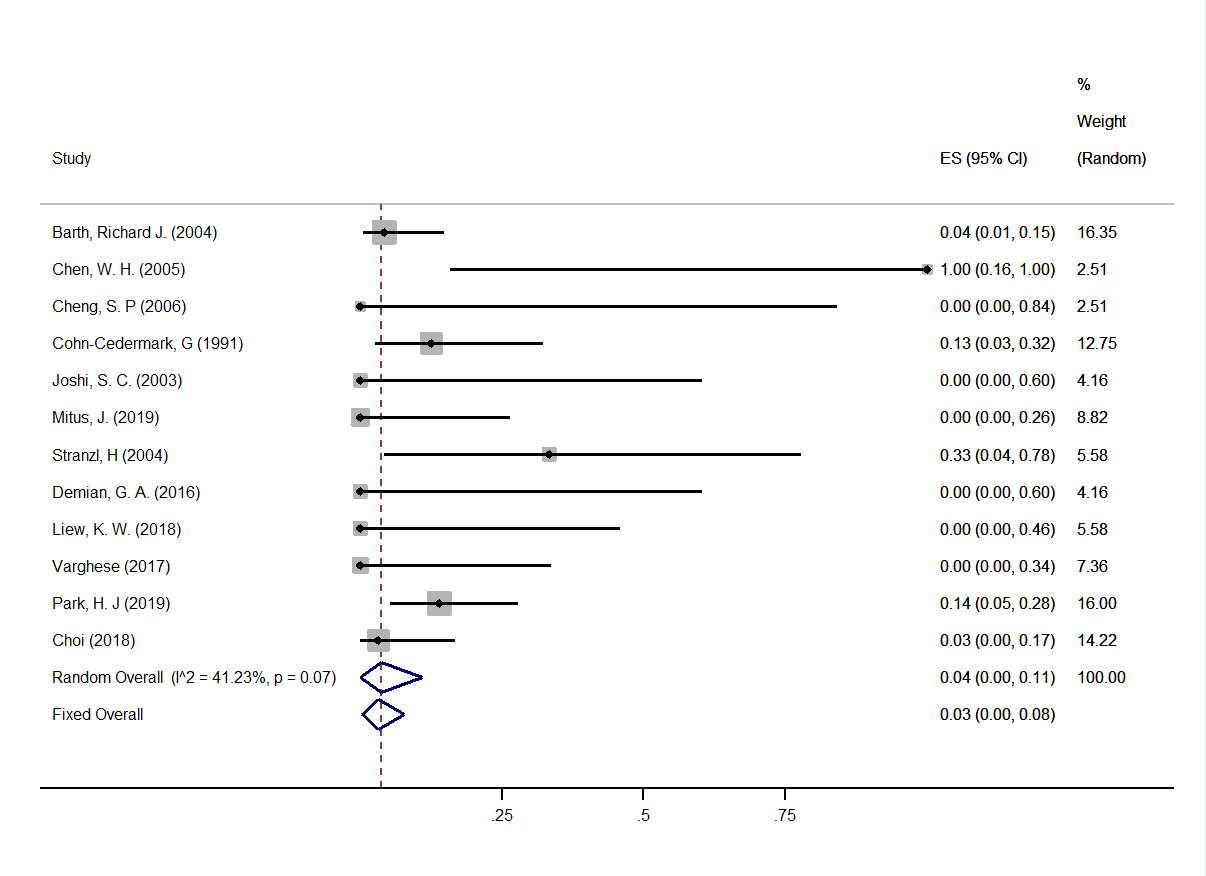

Supplement: Supplementary file 1 — Figure S1. Meta-analysis of metastasis rate of patients treated with radiotherapy (random model). (TIF 3097 kb) [file 12885_2019_5585_MOESM1_ESM.tif]

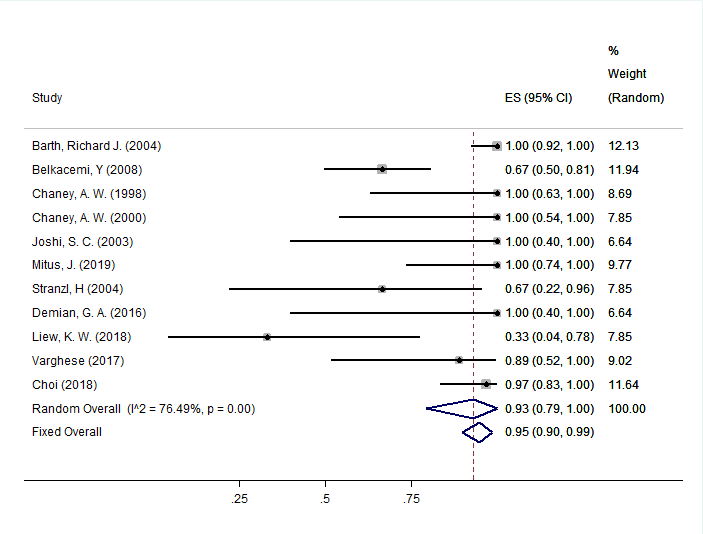

Supplement: Supplementary file 3 — Figure S2. Meta-analysis of disease-free survival rate of patients treated with radiotherapy (random model). (TIF 1101 kb) [file 12885_2019_5585_MOESM3_ESM.tif]

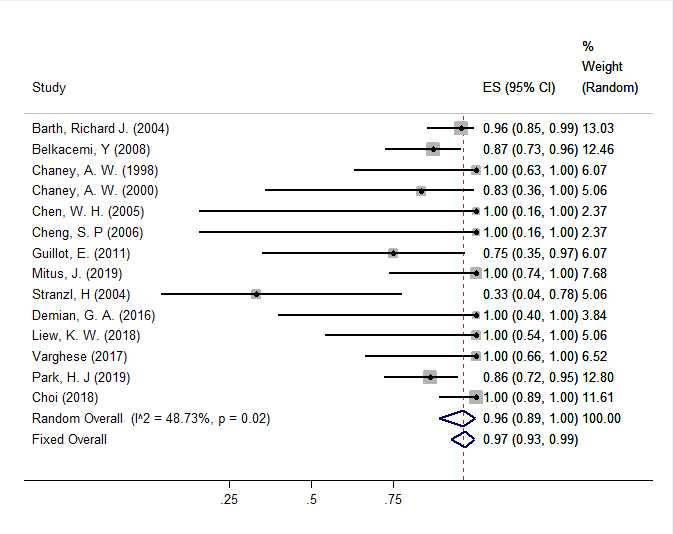

Supplement: Supplementary file 4 — Figure S3. Meta-analysis of overall survival rate of patients treated with radiotherapy (random model). (TIF 1054 kb) [file 12885_2019_5585_MOESM4_ESM.tif]
