# Supplementary material for: Adjuvant radiotherapy and chemotherapy for patients with breast phyllodes tumors: a systematic review and meta-analysis
Source: BMC Cancer. 2019 Apr 23;19:372. doi: 10.1186/s12885-019-5585-5 (PMC6480723; doi:10.1186/s12885-019-5585-5)
Supplement: Supplementary file 2 — Table S1. Quality assessment of the included studies. Table S2. Subgroup analysis of metastasis rate of radiotherapy. Table S3. Subgroup analysis of disease-free survival rate. Table S4. Subgroup analysis of overall survival rate. (ZIP 73 kb) [file 12885_2019_5585_MOESM2_ESM.zip › Supplementary Table1R2.docx]

**Table S1.** Quality Assessment of the included 12 studies

|  | Criteria 1 | Criteria 2 | Criteria 3 | Criteria 4 | Criteria 5 | Criteria 6 | Criteria 7 | Criteria 8 | Criteria 9 | Quality Rating |
| --- | --- | --- | --- | --- | --- | --- | --- | --- | --- | --- |
| Barth, Richard J. | Yes | Yes | Yes | Yes | Yes | Yes | Yes | Yes | Yes | Good |
| Belkacemi | Yes | Yes | Yes | Yes | Yes | Yes | Yes | Yes | Yes | Good |
| Chaney, A. W. | Yes | Yes | Yes | Yes | Yes | Yes | Yes | Yes | Yes | Good |
| Chaney, A. W. | Yes | Yes | Yes | Yes | Yes | Yes | Yes | Yes | Yes | Good |
| Chen, W. H. | Yes | Yes | Yes | Yes | Yes | Yes | Yes | Yes | Yes | Good |
| Cheng, S. P | Yes | Yes | Yes | Yes | Yes | Yes | Yes | Yes | Yes | Good |
| Cohn-Cedermark, G | Yes | Yes | Yes | Yes | Yes | Yes | Yes | Yes | Yes | Good |
| Demian | Yes | Yes | Yes | Yes | Yes | Yes | Yes | Yes | Yes | Good |
| Gnerlich, J. L | Yes | Yes | Yes | Yes | Yes | Yes | Yes | Yes | Yes | Good |
| Guillot, E. | Yes | Yes | Yes | Yes | Yes | Yes | Yes | Yes | Yes | Good |
| Joshi, S. C. | Yes | Yes | Yes | Yes | Yes | Yes | Yes | Yes | Yes | Good |
| Liew, K. W. | Yes | Yes | Yes | Yes | Yes | Yes | Yes | Yes | Yes | Good |
| Mitus, J. | Yes | Yes | Yes | Yes | Yes | Yes | Yes | Yes | Yes | Good |
| Park, H. J. | Yes | Yes | Yes | Yes | Yes | Yes | Yes | Yes | Yes | Good |
| Stranzl, H | Yes | Yes | Yes | Yes | Yes | Yes | Yes | Yes | Yes | Good |
| Varghese | Yes | Yes | Yes | Yes | Yes | Yes | Yes | Yes | Yes | Good |

Quality Assessment Tool for Case Series Studies recommended by U.S. Department of Health & Human Services

**Criteria:**

1. Was the study question or objective clearly stated?

2. Was the study population clearly and fully described, including a case definition?

3. Were the cases consecutive?

4. Were the subjects comparable?

5. Was the intervention clearly described?

6. Were the outcome measures clearly defined, valid, reliable, and implemented consistently across all study participants?

7. Was the length of follow-up adequate?

8. Were the statistical methods well-described?

9. Were the results well-described?
