# Supplementary material for: Adjuvant radiotherapy and chemotherapy for patients with breast phyllodes tumors: a systematic review and meta-analysis
Source: BMC Cancer. 2019 Apr 23;19:372. doi: 10.1186/s12885-019-5585-5 (PMC6480723; doi:10.1186/s12885-019-5585-5)
Supplement: Supplementary file 2 — Table S1. Quality assessment of the included studies. Table S2. Subgroup analysis of metastasis rate of radiotherapy. Table S3. Subgroup analysis of disease-free survival rate. Table S4. Subgroup analysis of overall survival rate. (ZIP 73 kb) [file 12885_2019_5585_MOESM2_ESM.zip › Supplementary Table2R2.docx]

**Table S2**. Sub-group analysis of metastasis rate of radiotherapy

| Characteristic | No. of studies | Recurrence rate (95%CI) | Heterogeneity | |
| --- | --- | --- | --- | --- |
|  |  |  | P | I^2^ (%) |
| Study size |  |  |  |  |
| < 20 | 8 | 0.04 (0.00-0.23) | 0.04 | 52.5 |
| ≥ 20 | 3 | 0.09 (0.04-0.17) | 0.05 | 28.1 |
| Follow-up |  |  |  |  |
| < 5yrs | 7 | 0.01 (0.00-0.06) | 0.62 | 0 |
| ≥ 5yrs | 4 | 0.12 (0.00-0.35) | 0.01 | 71.9 |
| Surgery type |  |  |  |  |
| BCS ≥ 60% | 5 | 0.09 (0.00-0.24) | 0.02 | 66.5 |
| BCS < 60% | 6 | 0.00 (0.00-0.10) | 0.43 | 0 |
| Age |  |  |  |  |
| < 45 | 6 | 0.06 (0.00-0.28) | 0.08 | 62.2 |
| ≥ 45 | 5 | 0.05 (0.00-0.14) | 0.20 | 33.1 |
| Tumor size |  |  |  |  |
| < 5cm | 2 | 0.02 (0.00-0.09) | - | - |
| ≥ 5cm | 8 | 0.06 (0.00-0.23） | 0.04 | 53.1 |
| Histologic Type |  |  |  |  |
| Malignant ≥ 30% | 6 | 0.07 (0.01-0.15) | 0.16 | 37.4 |
| Malignant < 30%  Margin  > 1cm ≥ 50%  > 1cm < 50% | 5  2  2 | 0.07 (0.00-0.45)  0.01 (0.00-0.09)  0.05 (0.00-0.23) | 0.05  -  - | 57.8  -  - |
| Positive < 10% | 3 | 0.07 (0.00-0.20) | - | - |
| Positive > 10% | 3 | 0.18 (0.00-0.88) | 0.01 | 77.9 |
