# Supplementary material for: Adjuvant radiotherapy and chemotherapy for patients with breast phyllodes tumors: a systematic review and meta-analysis
Source: BMC Cancer. 2019 Apr 23;19:372. doi: 10.1186/s12885-019-5585-5 (PMC6480723; doi:10.1186/s12885-019-5585-5)
Supplement: Supplementary file 2 — Table S1. Quality assessment of the included studies. Table S2. Subgroup analysis of metastasis rate of radiotherapy. Table S3. Subgroup analysis of disease-free survival rate. Table S4. Subgroup analysis of overall survival rate. (ZIP 73 kb) [file 12885_2019_5585_MOESM2_ESM.zip › Supplementary Table3R2.docx]

**Table S3.** Sub-group analysis of the disease-free survival rate

| Characteristic | No. of studies | DFS rate (95%CI) | Heterogeneity | |
| --- | --- | --- | --- | --- |
|  |  |  | P | I^2^ (%) |
| Study size |  |  |  |  |
| < 20 | 8 | 0.93(0.76-1.00) | 0.03 | 54.7 |
| ≥ 20 | 1 | 0.67(0.50-0.81) | - | - |
| Follow-up |  |  |  |  |
| < 5yrs | 4 | 0.96(0.79-1.00) | 0.27 | 23.8 |
| ≥ 5yrs | 5 | 0.83(0.57-0.99) | < 0.01 | 73.8 |
| Surgery type |  |  |  |  |
| BCS ≥ 60% | 2 | 0.63(0.46-0.78) | - | - |
| BCS < 60% | 7 | 0.98(0.89-1.00) | 0.48 | 0.00 |
| Age |  |  |  |  |
| < 45 | 6 | 0.93(0.76-1.00) | 0.06 | 53.7 |
| ≥ 45 | 3 | 0.75(0.23-1.00) | 0.01 | 82.9 |
| Tumor size |  |  |  |  |
| < 5cm | 2 | 0.63(0.47-0.78) | - | - |
| ≥ 5cm | 6 | 0.97(0.87-1.00） | 0.38 | 5.5 |
| Histologic Type |  |  |  |  |
| Malignant ≥ 30% | 3 | 0.91(0.66-1.00) | 0.11 | 54.7 |
| Malignant < 30% | 6 | 0.88(0.64-1.00) | 0.01 | 68.4 |
