# Supplementary material for: Adjuvant radiotherapy and chemotherapy for patients with breast phyllodes tumors: a systematic review and meta-analysis
Source: BMC Cancer. 2019 Apr 23;19:372. doi: 10.1186/s12885-019-5585-5 (PMC6480723; doi:10.1186/s12885-019-5585-5)
Supplement: Supplementary file 2 — Table S1. Quality assessment of the included studies. Table S2. Subgroup analysis of metastasis rate of radiotherapy. Table S3. Subgroup analysis of disease-free survival rate. Table S4. Subgroup analysis of overall survival rate. (ZIP 73 kb) [file 12885_2019_5585_MOESM2_ESM.zip › Supplementary Table4R2.docx]

**Table S4.** Sub-group analysis of overall survival rate

| **Characteristic** | **No. of studies** | **Recurrence rate (95%CI)** | **Heterogeneity** | |
| --- | --- | --- | --- | --- |
|  |  |  | **P** | **I^2^ (%)** |
| Study size |  |  |  |  |
| < 20 | 10 | 0.95(0.82-1.00) | 0.07 | 44.1 |
| ≥ 20 | 3 | 0.90(0.83-0.96) | 0.23 | 31.2 |
| Follow-up |  |  |  |  |
| < 5yrs | 9 | 0.94(0.81-1.00) | 0.04 | 49.9 |
| ≥ 5yrs | 4 | 0.93(0.85-0.92) | 0.44 | 0 |
| Surgery type |  |  |  |  |
| BCS ≥ 60% | 6 | 0.94(0.88-0.98) | 0.41 | 1.1 |
| BCS < 60% | 7 | 0.95(0.77-1.00) | 0.03 | 56.6 |
| Age |  |  |  |  |
| < 45 | 9 | 0.94(0.87-0.99) | 0.76 | 0 |
| ≥ 45 | 4 | 0.92(0.66-1.00) | 0.01 | 76.3 |
| Tumor size |  |  |  |  |
| < 5cm | 4 | 0.93(0.84-0.99) | 0.23 | 29.6 |
| ≥ 5cm | 8 | 0.96(0.82-1.00） | 0.05 | 50.7 |
| Histologic Type |  |  |  |  |
| Malignant ≥ 30% | 5 | 0.92(0.76-1.00) | 0.01 | 72.8 |
| Malignant < 30%  Margin  > 1cm ≥ 50%  > 1cm < 50%  Positive > 10%  Positive < 10% | 8  3  3  2  4 | 0.95(0.86-1.00)  0.95(0.81-1.00)  0.83(0.48-1.00)  0.93(0.80-1.00)  0.90(0.80-1.00) | 0.45  0.19  < 0.01  -  0.76 | 0  40.1  81.4  -  0 |
